# The Effect of Media Professionalization on Cognitive Neurodynamics During Audiovisual Cuts

**DOI:** 10.3389/fnsys.2021.598383

**Published:** 2021-01-28

**Authors:** Celia Andreu-Sánchez, Miguel Ángel Martín-Pascual, Agnès Gruart, José María Delgado-García

**Affiliations:** ^1^Department of Audiovisual Communication and Advertising, Neuro-Com Research Group, Universitat Autònoma de Barcelona, Barcelona, Spain; ^2^Serra Húnter Fellow; ^3^Technological Innovation, Instituto Radio Televisiòn Española (RTVE), Corporaciòn RTVE, Barcelona, Spain; ^4^Division of Neuroscience, University Pablo de Olavide, Sevilla, Spain

**Keywords:** neurocinematics, professionalization, expertise, connectivity, visual perception, film cuts

## Abstract

Experts apply their experience to the proper development of their routine activities. Their acquired expertise or professionalization is expected to help in the development of those recurring tasks. Media professionals spend their daily work watching narrative contents on screens, so learning how they manage visual perception of those contents could be of interest in an increasingly audiovisual society. Media works require not only the understanding of the storytelling, but also the decoding of the formal rules and presentations. We recorded electroencephalographic (EEG) signals from 36 participants (18 media professionals and 18 non-media professionals) while they were watching audiovisual contents, and compared their eyeblink rate and their brain activity and connectivity. We found that media professionals decreased their blink rate after the cuts, suggesting that they can better manage the loss of visual information that blinks entail by sparing them when new visual information is being presented. Cuts triggered similar activation of basic brain processing in the visual cortex of the two groups, but different processing in medial and frontal cortical areas, where media professionals showed a lower activity. Effective brain connectivity occurred in a more organized way in media professionals–possibly due to a better communication between cortical areas that are coordinated for decoding new visual content after cuts.

## Introduction

Professionalization in anything requires expertise along with a long-time training. As a result, experts have acquired, through experience, the perceptual skills to make fine discriminations (Klein and Hoffman, 1993). Professional athletes have extraordinary skills for learning complex visual scenes (Faubert, 2013). It has been previously proven that professionalization has an impact on cognitive neurodynamics in many brain areas. Event-related desynchronization (ERD) in alpha and beta frequency bands during action observation is sensitive to expertise in contemporary dance. In addition, looking at dance movements evokes desynchronization effects in professional dancers, but not in non-dancers (Orgs et al., 2008). The influence of motor expertise on action observation has also been proven with expert dancers (Calvo-Merino et al., 2005). Expert baseball players show differential activity in their post-task resting state consistent with motor learning, and functional differences found between expert and non-expert baseball players suggest variability in subcortical white-matter pathways (Muraskin et al., 2016). Taxi drivers show navigation-related structural changes in their hippocampi (Maguire et al., 2000, 2006). Expertise in aesthetics modulates cognitive processing and the response in reward-related brain areas (Kirk et al., 2008). The case of musicians may be one of the most studied. For example, it has been found that brain structures, including primary motor and somatosensory areas, differ between musicians and non-musicians (Gaser and Schlaug, 2003). Professional violinists exhibit higher activity in the primary auditory cortex during the execution of a musical piece compared with amateurs, suggesting an increased functional strength in the audio-motor associative connectivity (Lotze et al., 2003). Finally, musical expertise is related to altered functional connectivity during audiovisual integration (Paraskevopoulos et al., 2015).

Regarding audiovisual expertise, we have previously found that media professionalization has an impact in visual perception (Andreu-Sánchez et al., 2017b). Media professionals are watching screens steadily over time (taking concomitant decisions with a high level of attention), and decrease their eyeblink rate not only when watching media works, but also when looking at live events (Andreu-Sánchez et al., 2017a). Media professionals are constantly paying attention to cuts since those are one of the most common tools for audio-visual editors. Cuts segment narrative content, playing a critical role in the proper understanding of observed visual material (Zacks et al., 2010). Their use has been increasing in cinema in the last few decades, reducing the average length of shots (Cutting et al., 2011). Cuts interrupt the visual information that a movie is presenting to viewers, who are nevertheless not conscious of them, provoking a phenomenon called edit blindness (Smith and Henderson, 2008). Both the attentional level and the awareness of cuts depend on the edit construction. A chaotic and non-organized style of edition, plenty of cuts, decreases viewers' eyeblinks compared with a classical edition having fewer and more-organized cuts, and even more compared with a one-shot style without cuts (Andreu-Sánchez et al., 2017b). Since eyeblinks can be understood as attentional markers when watching movies (Nakano et al., 2009), it is important to note that cuts can have a big impact on the management of viewers' attention. Also, memory is better for information presented after related cuts compared with unrelated cuts between two unrelated scenes (Lang et al., 1993). But this event segmentation is linked not only to the style, but also to the meaningful changes in narrative situations (Zacks et al., 2010). In fact, movie viewers move their eyes based on the presented content (for example, to the current speaker in a depicted conversation), suggesting that despite the changes that cuts make in a visual scene, viewers tend to adapt their visual behavior to the content (Germeys and D'Ydewalle, 2007).

Event-related potentials (ERPs) after the cut are coherent with studies on early discrimination of visual stimuli (Thorpe et al., 1996). Activity flows from the primary visual zones to somatosensory and prefrontal areas (Andreu-Sánchez et al., 2018). Besides, the style of edition in which cuts are inserted affects viewers' perception. While cuts in chaotic and fast audiovisuals increase brain activity in the visual cortex, suggesting an increase of attentional scope, cuts in organized and continuous movies increase brain activity in the prefrontal cortex (Andreu-Sánchez et al., 2018), where higher processing areas are located. Interestingly, a study on a very concrete editing rule–the 180° rule, which says that two edited shots of the same event or action should not be filmed from different angles that violate spatial continuity–showed that differences in perception were not linked to visual attention but to sensorimotor activity (Heimann et al., 2016).

To detect the effect of media professionalization on the viewing of audiovisual cuts, here we have investigated differences in brain activity after the cut between media and non-media professionals. We addressed this goal by analyzing and comparing in the two groups the spontaneous eyeblink rate (SBR), the EEG brain activity, and brain connectivity.

## Materials and Methods

### Subjects

Thirty-six participants (30 males and 6 females) took part in this experiment. Eighteen were media professionals (mean age: 43.89 ± 8.79; 15 males and 3 females), and the rest were non-media professionals (mean age: 44.06 ± 7.53; 15 males and 3 females). All had normal or corrected-to-normal vision. For being part of the media professional group, subjects had to have a job related to video edition, including taking decisions related to media editing. Media professional participants were producers, assistant producers, cameramen, image controllers, documentalists, graphic designers, post-production editors, sports commentators, and video editors. Media professionals were mostly recruited from the Spanish Public Television (RTVE) installations in Barcelona. Non-media professionals were carefully chosen outside this criterion. They were individuals who did not make decisions related to media editing and audio-visual cuts in their work. Non-media professional participants were journalists with no media editing responsibilities, computer specialists, administrative and management assistants, telecommunication engineers, electronics technicians, stylists, specialists in prevention of occupational risks, and executive producers with no artistic profile. Subjects did not receive any economic compensation for participating in this investigation.

### Ethics Statement

After a detailed explanation of the study, written informed consent was signed by all participants. Experiments were carried out following guidelines, procedures, and regulations for human research of the University Autònoma de Barcelona and approved by its Ethics Commission for Research with Animals and Humans (CEEAH; file number 2003). All experimental sessions were performed at the Neuro-Com Laboratory located at the Spanish Public Television Institute (IRTVE) in Sant Cugat del Vallès, Barcelona, Spain.

### Stimuli and Experimental Design

For the experimental sessions, we created four visual stimuli with the same duration (198 s) and narrative content, but with different formats. All stimuli had the same visual content with different style of edition. The action consisted of a man who entered a room with a black background, sat at a desk, juggled with three balls, opened a laptop, looked up some information in books, wrote something in the laptop, closed it, ate an apple, looked directly into camera, and left the room. Stimulus 1 was a one-shot movie; stimulus 2 was a movie edited according to classical Hollywood rules (Bordwell et al., 1985); stimulus 3 was a movie edited according to MTV style (Bordwell, 2002); and stimulus 4 was a live performance. All stimuli were randomly presented to all participants [see Andreu-Sánchez et al. (2017b) for details]. The order of presentation of the four stimuli was randomized with the 24 possible combinations to avoid the impact of sensory adaption and effect of fatigue. The presentation of each stimulus was preceded of 30 s of a black screen. Stimulus 2 presented 33 cuts and stimulus 3 presented 79 cuts. Since the interest of this study was to analyze differences in media professionals compared with non-media professionals while viewing audiovisual cuts, for the present paper we just analyzed data collected during the presentation of stimuli 2 and 3. A total of 4,032 potential cuts (112 cuts per participant) form the sample used in this investigation, before discarding bad data. The task for participants consisted in just watching the visual stimuli.

Video stimuli were presented on a 42-inch HD LED display (Panasonic TH-42PZ70EA, Panasonic Industry Iberia, Madrid, Spain), and participants were placed 150 cm in front of the screen. Stimuli were presented and synchronized with the Paradigm Stimulus Presentation (Perception Research System Incorporated) and the NIC Offline software (Neuroelectrics, Barcelona, Spain).

### Data Acquisition

Continuous EEGs were recorded using a wireless system (Enobio®, Neuroelectrics), with 20 electrodes placed according to the International 10–20 system [O1, O2, P7, P3, Pz, P4, P8, T7, C3, Cz, C4, T8, F7, F3, Fz, F4, F8, Fp1, Fp2, and an external electrode used for electrooculogram (EOG) recording] referenced to electronically linked mastoid electrodes [see Martín-Pascual et al. (2018) for details]. The EOG electrode was positioned vertically at the infraorbital ridge and the lower outer canthus of the left eye and was used to monitor eyeblinks. Data were sampled at 500 Hz. In order to have a good quality of EEG signal, participants were asked to wash their hair before attending the session and to avoid any chemical product (such as a hair spray or similar) on it. An HD-video camera (Sony HDR-GW55VE, Sony Corporation España, Barcelona, Spain) recorded at 25 frames/s participants' faces with a close-up for contrasting their eye movements and eyeblinks during the analysis of the eyeblink rate.

### Data Analysis

#### Spontaneous Eyeblink Rate

For analyzing the SBR of each participant, we processed original data with Brainstorm open-source version 3 running on MATLAB R2013a (The Mathworks Inc., Natick, MA, USA) under a MacOS version 10.9.5 (Apple Inc., Cupertino, CA, USA). We filtered the signal from 0.5 to 3 Hz, applied Brainstorm's eyeblink detector, and manually checked the collected results (Tadel et al., 2015). We analyzed original data from Fp1, Fp2, and EOG electrodes. Following Nakano and Kitazawa (Nakano and Kitazawa, 2010), we also checked the results collected with the HD-video camera of participants' faces. To do so and using UNIX Time for synchronization purposes, we manually played the HD-video frame by frame while looking at the time of each eyeblink detected by the Brainstorm's detector and checking that it was in fact an eyeblink. We did not find differences with the other recording procedures. We quantified the rate of SBR/min. We compared two different variables: group (SBR of media vs. non-media professionals) and cut (SBR 1 s after the cut vs. SBR within the rest of the stimuli) through Whitney Rank Sum Tests and *t-*tests. To determine normality of data we carried out a Shapiro-Will test. The statistical analysis was carried out with Sigmaplot 11.0 (Systat Software Inc., San Jose, CA, USA).

#### Event-Related Potentials, Event-Related Spectrum Perturbances, and Power Spectrum

We first analyzed EEG data to obtain ERPs, event-related spectrum perturbances (ERSPs), and power spectrum. We created a study in EEGLAB (Delorme and Makeig, 2004) open-source version 2019_1, running on MATLAB_R2020a (The Mathworks Inc.) under a MacOS High Sierra version 10.13.6 for comparisons and statistical analysis. Previously, for pre-processing the data, we had used EEGLAB 15.3 on MATLAB R2013a under a MacOS version 10.9.5. We used a spherical BESA® template for channel location. We band-passed data with 0.5 and 40 Hz. We processed the data in 1,500 ms windows (from 500 ms before each cut to 1,000 ms after it). For rejecting artifacts, bad channels, and wrong data, we used visual inspection and the ADJUST plug-in (Mognon et al., 2010) for EEGLAB, after applying independent component analysis. To locate dipoles, we used the DIPFIT plug-in (Oostenveld and Oostendorp, 2002). We analyzed ERP signatures for different periods of the 1,500-ms epochs (−500 to 1,000 ms) through a three-way analysis of variance (ANOVA) taking as factors the following: (1) professionalization (media professionals, non-media professionals); (2) scalp area, with three scalp areas (frontal area with F7, F3, Fz, F4, and F8 electrodes; central area with C3, Cz, and C4 electrodes; and parieto-occipital area with P7, P3, Pz, P4, P8, O1, and O2 electrodes); and (3) latency, with three time windows (400–600 ms after the cut, 600–800 ms after the cut, and 800–1,000 ms after the cut). We approached ERSPs in somatosensory area through an unpaired *t-*test between the electrodes of this specific scalp area. For power spectra analysis, we analyzed the alpha band (8–12 Hz) for the same three scalp areas through Mann-Whitney Rank Sum tests. Analyses were done to contrast differences between media professionals and non-media professionals. Before approaching tests, we checked normality of data with Shapiro-Will normality test. Statistical analysis was carried out with EEGLAB Statistics Toolbox and Sigmaplot 11.0 (Systat Software Inc.).

#### Brain Connectivity

We approached brain connectivity from functional and effective connectivity analysis. We used clean datasets resulting from the described filtering and used the MATLAB toolbox HERMES (Niso et al., 2013) for brain connectivity analysis.

Functional connectivity can be defined as the temporal correlation of a neurophysiological index measured in different brain areas (Friston et al., 1993). It implies a temporal dependency of neuronal activation patterns of anatomically separated brain regions (van den Heuvel and Hulshoff Pol, 2010). For analyzing functional connectivity, we used the method of phase-locking value (PLV), which detects synchrony in a precise frequency range between two recording sites (Lachaux et al., 1999). PLV uses responses to a repeated stimulus (in this case, cuts) and looks for latencies at which the phase difference between the signals varies little across trials (phase-locking). Given two series of signals (x and y), and a frequency of interest (f), the PLV method computes a measure of phase-locking between the components of x and y at frequency f, for each latency (Lachaux et al., 1999). So, PLV makes use only of the relative phase difference and is defined (Niso et al., 2013) as:

(1) $$\begin{array}{l} PLV=\;\left|\langle \;e^{i\Delta \phi }rel^{\left(t\right)}\rangle \;\right|=\;\left|\frac{1}{N}\;\overset{N}{\underset{n-1}{\sum }}e^{i\Delta \phi }rel^{\left(t_{n}\right)}\right|\\ =\;\sqrt{{\langle cos\Delta \phi_{rel}\left(t\right)\rangle }^{2}+{\langle sin\Delta \phi_{rel}\left(t\right)\rangle }^{2}} \end{array}$$

where <.> indicates time average. Δϕ_rel_ = ϕ_x_ – ϕ_y_, where ϕ is the phase calculated from the Hilbert transform of x and y signals.

We computed the averaged connectivity of PLV in the 18 non-media professional participants and in the 18 media professional participants, with 100 surrogates of the original data, and compared functional connectivity between the two groups in theta (4–8 Hz), alpha (8–12 Hz), low beta (12–20 Hz), high beta (20–28 Hz), and low gamma (28–40 Hz). For the statistical analysis, we applied a *t-*test with multiple comparisons with false discovery rate Type 1 (q = 0.1) to the data with 100 surrogates. We looked for significant differences (*p* < 0.05) between groups.

Effective connectivity can be understood as the simplest experimental time-dependent circuit that replicates the timing relationships between the recorded sources (Aertsen and Preißl, 1991). It studies the influence that one neural system exerts over another (Friston, 1994). For analyzing effective connectivity here, we used classical linear Granger Causality (GC) (Granger, 1969). The concept behind GC is that for two simultaneously measured signals x(t) and y(t), if one can predict the first signal better by incorporating the past information from the second signal than by using only information from the first one, then the second signal can be called causal to the first (Wiener, 1956; Granger, 1969; Niso et al., 2013). The argument is that when x influences y, then if you add past values of x(t) to the regression of y(t), an improvement on the prediction will be obtained. GC from y to x (predicting x from y) can be defined (Niso et al., 2013) as:

(2) $$\begin{array}{l} GC_{y\to x}=ln\;(\frac{V_{\;x|\bar{x}}}{V_{\;x|\bar{x},\;\bar{y}}}) \end{array}$$

We computed the average connectivity of GC in the 18 non-media professional participants and in the 18 media professional participants after the cut (0–1,000 ms), with a baseline (−500 to 0 ms). We did 100 surrogates, with a false discovery rate correction (FDR) of Type 1 (q = 0.1), MaxDistance 1.5. We did a *t-*test to reveal significant differences (*p* < 0.05) between groups and plotted them.

For both types of connectivity analysis, we obtained binary outcomes for connectivity relations. Non-significant and significant relations (*p* < 0.05) were obtained based on this binary approximation among all pairs of electrodes.

More information about the implementation of these indices in HERMES is available elsewhere (Niso et al., 2013).

## Results

### Spontaneous Eyeblink Rate

**Figure 1:**
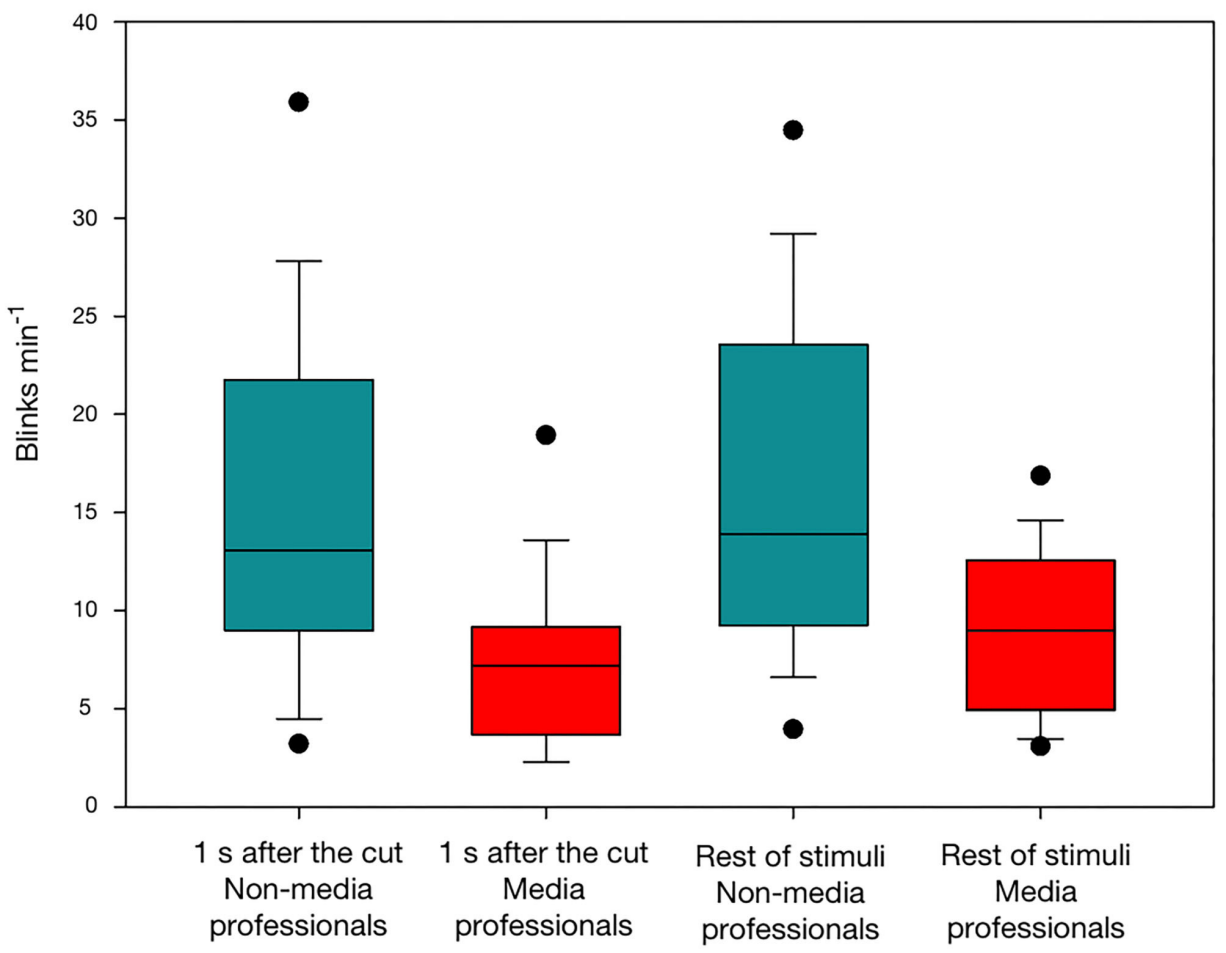
SBRs in non-media (red) and media (turquoise) professionals, during the first second after a cut and during the rest of video stimuli without cuts.

We identified substantial differences in the SBR related to media professionalization. Firstly, we obtained the mean SBR in the first second following the cut. While, among all participants, the mean SBR after the cut was 11.07 ± 7.66 min^−1^, in non-media professionals the mean SBR after the cut was 14.94 ± 8.43 min^−1^, and in media professionals it was 7.21 ± 4.25 min^−1^. The statistical comparison showed significant differences between the two groups after the cut (Mann-Whitney U Statistic = 60.5, T = 434.5, *n* = 18, *p* = 0.001, Mann-Whitney Rank Sum Test). Then, we obtained the mean SBR for the rest of the stimuli. For all participants, the mean SBR was 12.33 ± 7.61 min^−1^. In non-media professionals, the mean SBR was 15.79 ± 8.72 min^−1^, and in media professionals 8.87 ± 4.23 min^−1^. We also found, as expected, statistically significant differences between groups for the rest of the stimuli (Mann-Whitney U Statistic = 79.5, T = 415.5, *n* = 18, *p* = 0.009, Mann-Whitney Rank Sum Test). In a previous study (Andreu-Sánchez et al., 2018), we found that a cut decreases eyeblink rate in viewers during the second following it. Here, we wondered if those differences would be different depending on media professionalization. We found that they are. In media professionals, cuts have a greater impact with a decrease of SBR [t_(17)_ = −2.99, *p* = 0.008, paired *t-*test], while cuts seem not to have such an impact in non-media professionals [t_(17)_ = −1.14, *p* = 0.269, paired *t-*test]. These results suggest that, in line with previous studies (Andreu-Sánchez et al., 2017a), there is a media professionalization effect in visual perception of media contents (Figure 1).

### Event-Related Potentials and Event-Related Spectrum Perturbances

**Figure 2:**
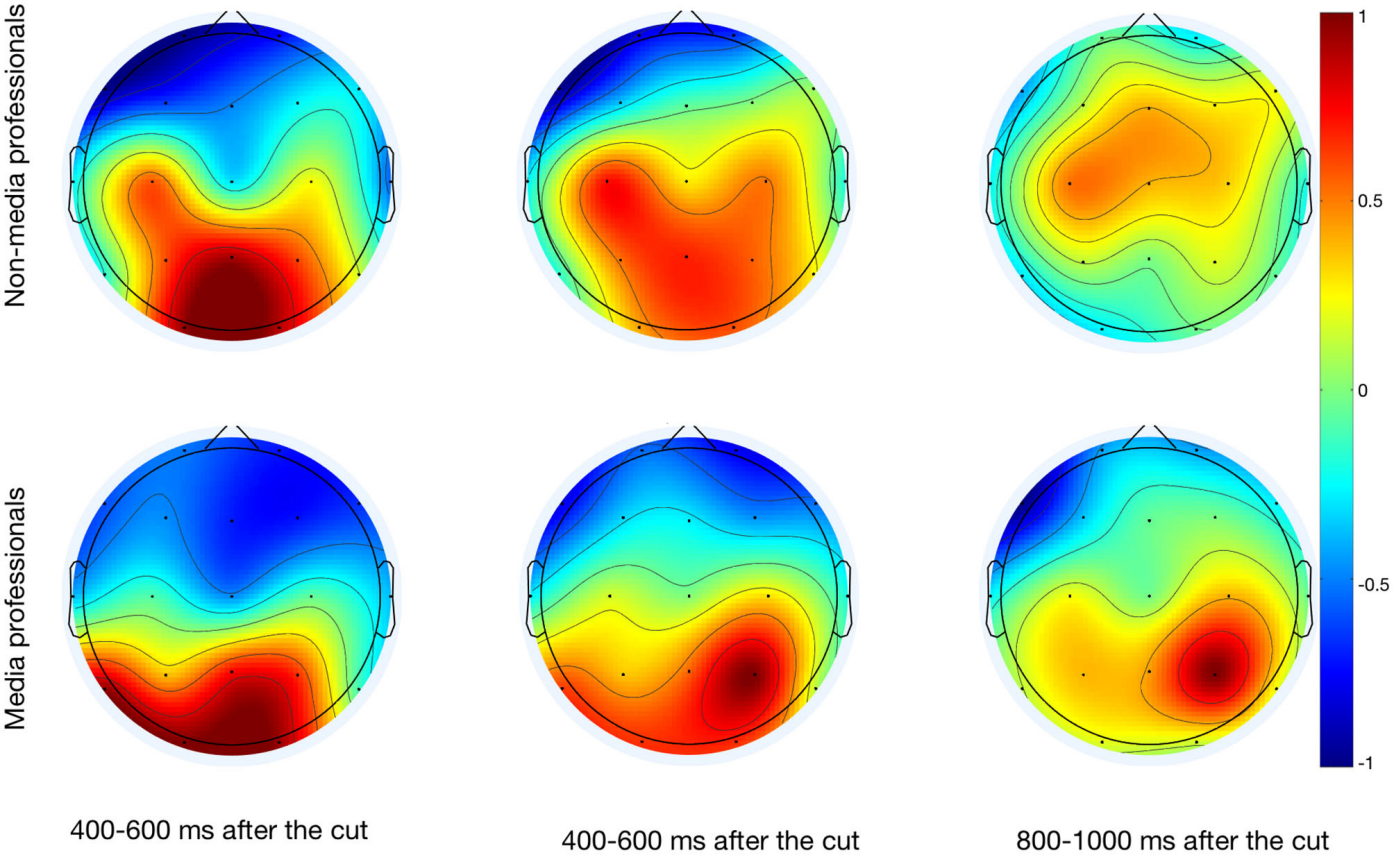
Scalp map after the cuts of the three analyzed time windows (400–600, 600–800, and 800–100 ms) of media and non-media professionals.

We found an effect of media professionalization in ERPs during the viewing of audiovisual cuts. As previously reported (Andreu-Sánchez et al., 2018), we found significant effects related to scalp area [F_(2, 306)_ = 32.367; *p* < 0.001] and scalp area x latency [F_(4, 306)_ = 3.647; *p* = 0.006]. Interestingly, here we also found an effect of professionalization and scalp area [F_(2, 306)_ = 4.822; *p* = 0.009]. With a Holm-Sidak method for *post-hoc* pairwise multiple comparisons, we found significant differences between groups in frontal [t_(0.264)_ = 2.034, *p* = 0.043] and central [t_(0.724)_ = 5.587, *p* < 0.001], but not occipital areas (Figure 2).

**Figure 3:**
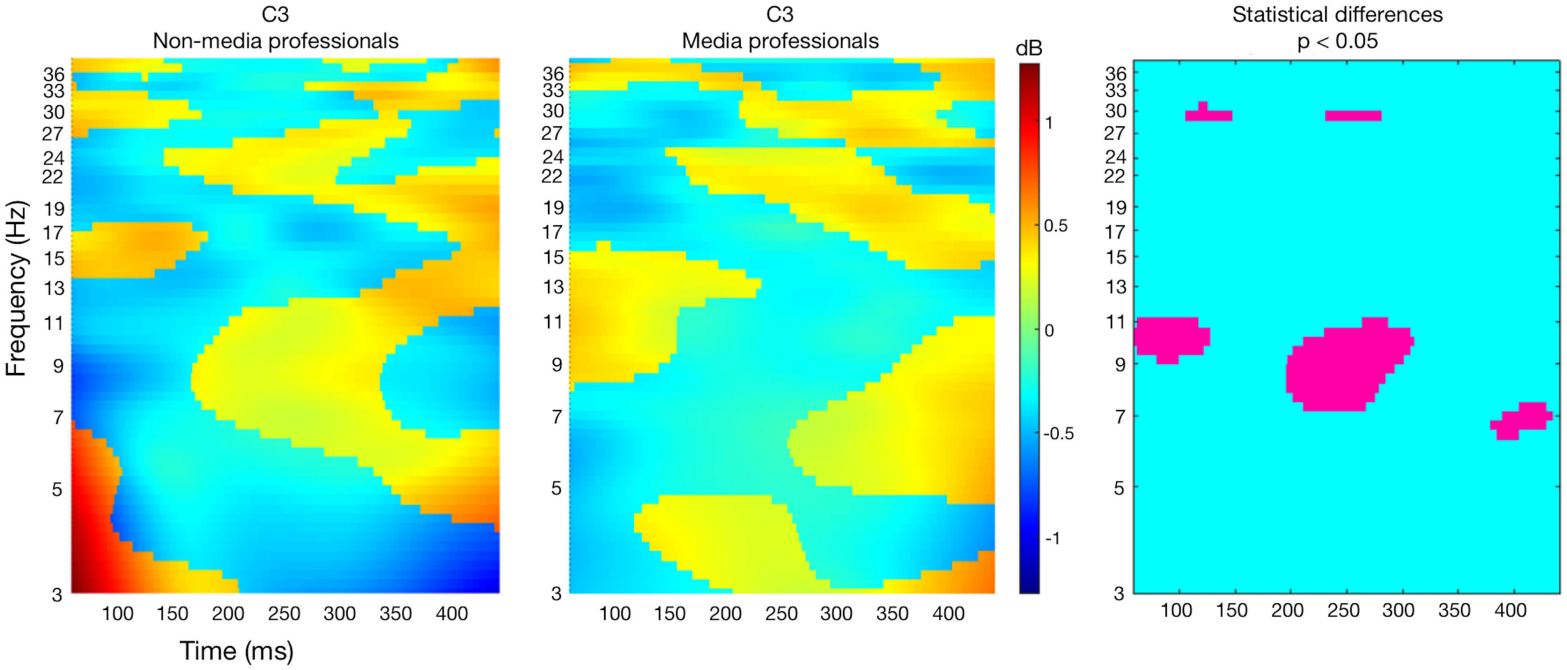
ERSPs in non-media (left) and media (middle) professionals, in electrode C3. The right image shows in pink the statistically significant differences between the two groups (unpaired *t-*test with no correction method for multiple comparison, *p* < 0.05).

Based on previous studies linked to somatosensory area differences related to expertise (Gaser and Schlaug, 2003; Orgs et al., 2008), we looked at ERSPs in that specific area, and found that there were differences in C3 (Figure 3). Media professionals showed greater activity between 7 and 11 Hz just after the cut, and a lower activity at those frequencies between 200 and 300 ms after the cut. This may be linked to a mu rhythm desynchronization, understood as the attenuation of power in the alpha band recorded over central scalp locations as a reflection of a motor cortex activity (Pfurtscheller, 1977; Pfurtscheller and Lopes da Silva, 1999; Debnath et al., 2019). Differences found here could depend on the media professionalization expertise. No such differences were found in Cz or C4, but this would be coincident with non-symmetric and contralateral sensorimotor activity (McFarland et al., 2000).

### Alpha Band

It has been reported that cuts provoke an increase of alpha power in parieto-occipital electrodes (Andreu-Sánchez et al., 2018). However, here we did not find statistically significant differences in the alpha band (8–12 Hz) between media and non-media professionals in any of the studied areas either before or after the cut: frontal area, before the cut (Mann-Whitney U = 131, T = 364, *n* = 18, *p* = 0.335, Mann-Whitney Rank Sum Test); frontal area, after the cut (Mann-Whitney U = 156, T = 339, *n* = 18, *p* = 0.862, Mann-Whitney Rank Sum Test); somatomotor area, before the cut (Mann-Whitney U = 158, T = 329, *n* = 18, *p* = 0.912, Mann-Whitney Rank Sum Test); somatomotor area, after the cut (Mann-Whitney U = 145, T = 316, *n* = 18, *p* = 0.602, Mann-Whitney Rank Sum Test); parieto-occipital area, before the cut (Mann-Whitney U = 132, T = 363, *n* = 18, *p* = 0.351, Mann-Whitney Rank Sum Test); and parieto-occipital area, after the cut (Mann-Whitney U = 126, T = 369, *n* = 18, *p* = 0.261, Mann-Whitney Rank Sum Test).

### Brain Connectivity

#### Phase-Locking Value

**Figure 4:**
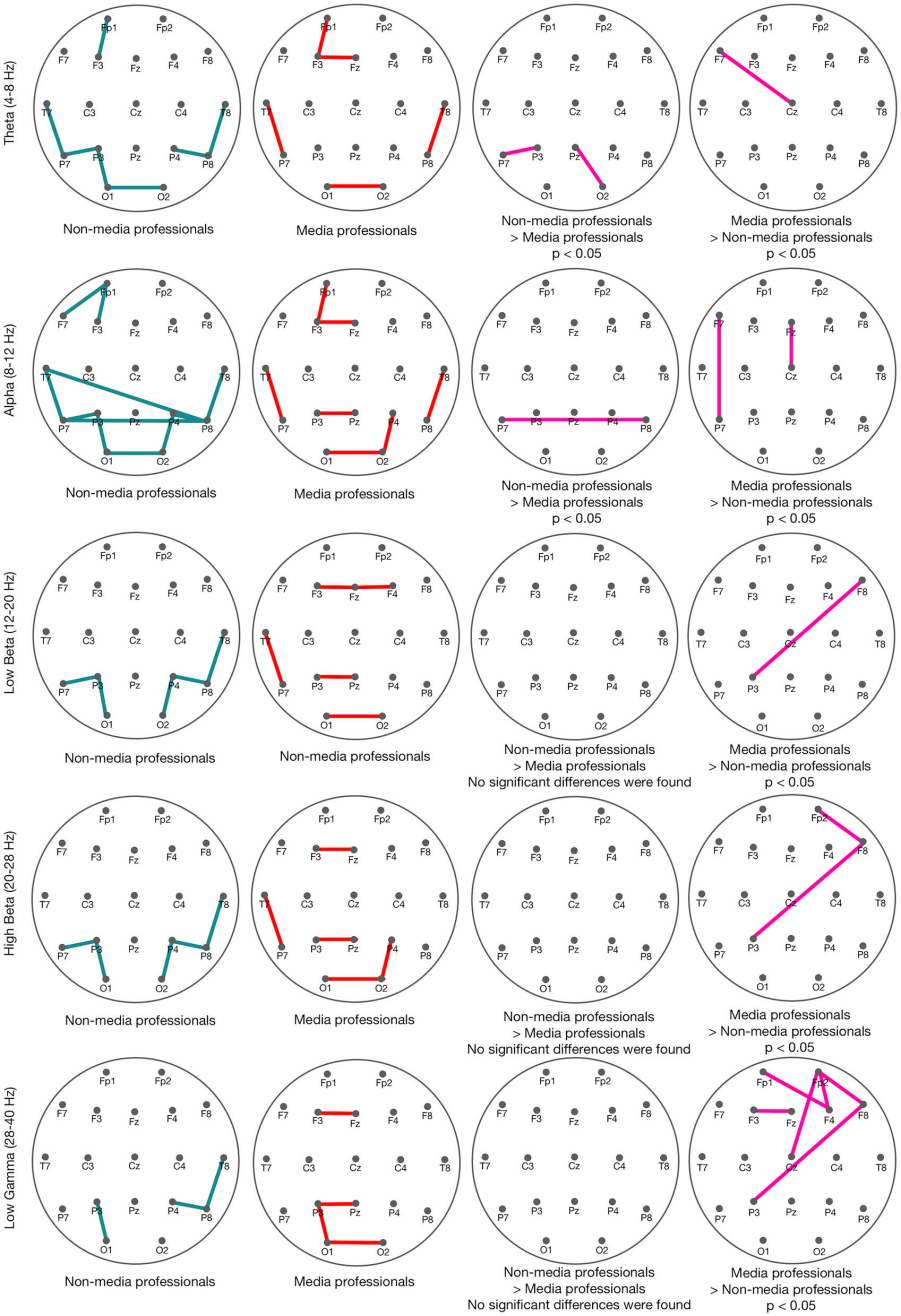
PLVs in non-media (turquoise) and media (red) professionals in different spectral bands: theta (4–8 Hz), alpha (8–12 Hz), low beta (12–20 Hz), high beta (20–28 Hz), and low gamma (28–40 Hz).

We found some significant differences between the two groups in functional connectivity in all the studied bands after the cut (Figure 4). In the theta band (4–8 Hz), we found some minor greater connectivity in non-media professionals in P7-P3 and Pz-O2, and in media professionals between Cz and F7. This suggests a higher functional connectivity in the theta band in parieto-occipital areas for non-media professionals and in prefrontal area for media professionals. In the alpha band (8–12 Hz), we also found a higher functional connectivity in occipital area in non-media professionals, while media professionals showed greater connectivity in central-frontal areas connection (Cz-Fz) and left parietal-frontal connection (P7–F7). We analyzed the beta band for low-beta (12–20 Hz) and high-beta (20–28 Hz) components. We found that in both cases, media professionals presented a higher functional connectivity between crossed sources (P3–F8), while non-media professionals did not show a higher PLV index at any point. In low gamma (28–40 Hz), we found that media professionals had a higher functional connectivity, mostly in frontal areas (F3, Fz, Cz-Fp2, F4-Fp1, F4-Fp2, F8-Fp2), and, again, a crossed flow (P3-F8). Non-media professionals did not show a higher PLV index at any source in low gamma.

#### Granger Causality

**Figure 5:**
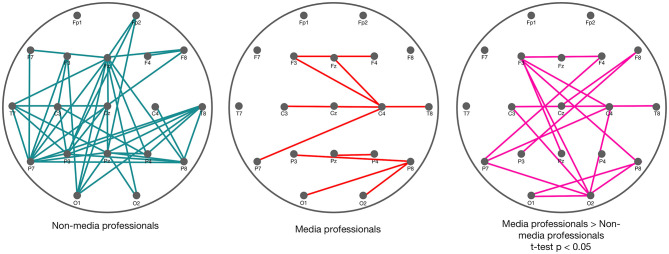
Granger causality in non-media (left) and media (middle) professionals, after the cut (0–1,000 ms, with a baseline of −500 ms before the cut), with significance level based on 100 surrogates. On the right, in pink, the statistically significant differences between the two groups (*t-*test, *p* < 0.05). Those results correspond to the higher GC indices in media professionals compared with the non-media group. No higher GC indices in non-media professionals were found.

With the aim of comparing effective connectivity between media and non-media professionals after the cut, we analyzed Granger causality. We obtained some significant differences in connectivity between groups (Figure 5). According to our results, non-media professionals show a more dispersed GC index in their brain activity, while media professionals' GC connectivity is much more concise since it is mostly concentrated in visual cortex, somatomotor, and frontal areas. And there is statistically significant higher activity in media professionals than in non-professionals in all three of those areas: occipital, medial and frontal.

## Discussion

In videos and movies, cuts provoke an artificial interruption of the narrative content. They are constantly used to present different visual information through different shots and visual compositions. A film is a stream of edited moving images consisting of hundreds or thousands of individual camera shots patched together (Heimann et al., 2016). But regardless of the several times per minute that cuts are present in a film, viewers are rarely aware of them, due to the so-called edit blindness (Smith and Henderson, 2008). Previous studies showed that cuts inhibit viewers' eyeblink rate (Andreu-Sánchez et al., 2017b, 2018). However, here we found that this happens with a clearer impact on media professionals, suggesting that this group is more sensitive to cuts. The rate of blinking has been proven to decrease when a cognitive operation is performed (Holland and Tarlow, 1975; Wong et al., 2002). Some studies have shown that watching a film reduces viewers' eyeblink rate (Patel et al., 1991; Andreu-Sánchez et al., 2017a), and it has been proposed that this decrease may be a strategy to minimize the loss of visual information (Nakano et al., 2009). The constant presentation of new visual information by cuts may be related to the decreased blink rate in viewers. And the fact that media professionals decrease their eyeblink rate after cuts significantly more than do non-media professionals may be related to the idea that experts can see what is not there and gain the ability to visualize how a situation has developed and to imagine how it is going to turn out (Klein and Hoffman, 1993). Maybe media professionals can better manage the fact that cuts are going to be followed by new visual information that needs to be decoded, and thereby avoid the loss of information that blinks entail, while non-media professionals are not so aware of cuts, showing a greater edit-blindness.

The context in which a cut is inserted and the situation to which a viewer pays attention affect visual perception (Zacks et al., 2010; Heimann et al., 2016). It is logical to think that cuts trigger an increase of activity in visual cortex that flows toward medial and frontal areas (Andreu-Sánchez et al., 2018) as other visual stimuli do (Thorpe et al., 1996). But since top-down directionality has been recently reinforced (Cheron et al., 2014; Halgren et al., 2019) it is important to keep researching about neuronal flows when watching audiovisual works. To date, using EEG signals for estimating the directionality of the neuronal flows is debated (Nolte et al., 2008) so further research should be done to complete this work. Here, we found that there are differences between media and non-media professionals in frontal and medial areas, while not in the visual cortex. In those frontal and medial zones non-experts presented a higher activity consistent with previous studies (Hill and Schneider, 2006). Apparently, cuts start the same (or a very similar) activation of basic brain processing of the new visual information presented, but how that visual content is managed by the two groups differs afterwards. In most cases, non-media professionals require a higher level of activity to process the visual information. One of the most interesting differences was found in the somatosensory area in C3. Media professionals showed a greater decrease of electrical activity at around 200–300 ms after the cut in C3, and this may be related to mu rhythm suppression during the observation of actions performed by other persons (Gastaut and Bert, 1954; Muthukumaraswamy et al., 2004); but in this case mu rhythm would also be affected by the expertise of the observer. Since previous studies revealed that mu suppression is sensitive to degree of familiarity (Oberman et al., 2008), it could be thought that media professionalization would bring a greater familiarity with media editing. Moreover, it is interesting to note that according to our results, the cut–and maybe other formal characteristics of the stimuli–could also affect mu rhythm suppression during the watching of videos. Our results invite us to keep researching on event-related synchronization (ERS) and desynchronization (ERD) in mu rhythm not only when watching motor activities in videos (Rayson et al., 2016) but also when approaching the language of film (Heimann et al., 2014). This is something that should be further investigated with aimed works for better understanding.

The alpha band has been linked with changes in attention (Reeves et al., 1985; Klimesch et al., 1998; Aftanas and Golocheikine, 2001; Sauseng et al., 2005). Following on from the difference of eyeblink rate found here between groups, and since eyeblinks are understood as markers of the attentional state of subjects (Ponder and Kennedy, 1927; York et al., 1971), we were interested in looking for differences in alpha frequencies. However, we did not observe significant differences in alpha band in any of the studied areas. It has been found that alpha frequencies influence whether a visual stimulus reaches awareness (Mathewson et al., 2009). Since we did not ask participants if they were aware or not of cuts, this connection should be further studied. While the role of beta oscillations in visual processing and attention is less clear (McCusker et al., 2020) further studies could approach if beta band in media professionals could be linked with the attention to visual stimuli (Michalareas et al., 2016). Since it has been hypothesized that beta oscillations may be stronger if the maintenance of the status quo is predicted than if a change is expected (Engel and Fries, 2010), it would be interesting learning about expectance to change while watching audiovisual works by the group of media professionals.

Despite the problem of the volume conduction for stablishing true synchrony (Lachaux et al., 1999) or coherency (Nolte et al., 2004) through biological tissue toward measurement sensors (scalp electrodes in this study), here dynamic measurements of functional and effective connectivity were approached through the PLV and the GC index, respectively. PLV is, of the many phase synchronization measurements available in the literature, one of the most used (Lachaux et al., 1999). It evaluates functional connectivity through the instantaneous phase difference of the signals under the hypothesis that connected areas generate signals whose instantaneous phases evolve together (Bruña et al., 2018). Cuts cause a synchronization effect (Andreu-Sánchez et al., 2018), but results reported here suggest that media and non-media professionals show different functional connectivity when watching cuts. Although those differences are discrete in most of the analyzed frequency bands (theta, alpha, and low and high beta), the general trend found here is a higher synchronization flow between posterior and frontal areas in media professionals. This suggests differences in functional connectivity related to the expertise in media. Apparently, media professionals would increase this kind of connectivity after viewing cuts, compared with non-professionals. This could be related to higher or faster conscious processing of the visual information after the cuts, or to a possibly lower edit blindness (Smith and Henderson, 2008) in this group. Our results suggest that media professionals would increase the connectivity between primary perceptive areas (such as the visual cortex) and more-cognitive processing areas (such as the prefrontal area). But the most-significant differences found between the two groups were in the low gamma band (28–40 Hz). According to our results, media professionals showed greater functional connectivity related to the frontal area in this frequency band. This may reflect differences in a participant's current state of attention or expectation (Hanslmayr et al., 2007) and could be related to the tendency of gamma to increase during novel stimuli (von Stein et al., 2000).

As already explained, we approached differences in effective connectivity between groups through GC. It is presented as a framework to quantify the asymmetric causal interactions between regions of the cortex and brain states (Sánchez-Campusano et al., 2011; Courellis et al., 2017). This measurement is fundamental for describing observed data in terms of directed functional interactions (Seth et al., 2015). Here, we found differences in effective connectivity between the two groups in occipital, parietal, medial, and frontal areas. Overall, media professionals showed high GC indices in three independent areas: occipital, medial, and frontal. It has been possible to distinguish GC in the activity within those areas. However, in non-media professionals, the GC found was much less organized and the connectivity was mostly within all different scalp areas (see Figure 5). This suggests that effective connectivity may occur in a more organized way in media professionals due to a better connectivity within areas that are coordinated for decoding new visual information after cuts. We found that the connectivity originating in those three areas (corresponding with visual cortex, somatomotor area, and frontal area) was stronger in media professionals. This is coincident with previous studies that attributed stronger connectivity in professionals compared with control groups to the learning undertaken because of their expertise (Sreenivasan et al., 2017), and that suggested an increased neural efficiency in the brain of highly skilled individuals (Bernardi et al., 2013). The fact that that higher connectivity in media professionals occurs in visual cortex could be related to the importance of that area for visual processing in their daily work. Something similar would occur in the frontal area, where higher cognitive processing of the visual information may be happening. Nevertheless, further studies would be needed to determine whether relevant differences between the two groups in somatosensory cortex could shed more light on this specific brain area, so many times connected to mu rhythm desynchronization and its possible relation to the mirror neuron system (Muthukumaraswamy et al., 2004; Pfurtscheller et al., 2006).

There are dozens of experiments tracking learning or expert performance. Patterns are beginning to emerge showing that learning and skilled performance produce changes in brain activation depending on the brain structure and the nature of the skill being learned (Hill and Schneider, 2006). The present study extends other studies of expertise showing that expertise and professionalization impact on cognitive neurodynamics (Bilalić, 2017). Learning how media professionals approach the perception of audiovisual works' may be useful in those areas that use screened content for training specific capabilities, such as aeronautics, some professional sports, or even basic education, among others.

## Data Availability Statement

The raw data supporting the conclusions of this article will be made available by the authors, without undue reservation.

## Ethics Statement

The studies involving human participants were reviewed and approved by Ethics Commission for Research with Animals and Humans (CEEAH). Universitat Autònoma de Barcelona. The patients/participants provided their written informed consent to participate in this study.

## Author Contributions

CA-S, MÁM-P, AG, and JMD-G experimental design and wrote the article. CA-S and MÁM-P carried out experiments and data analysis. All authors contributed to the article and approved the submitted version.

## Conflict of Interest

The authors declare that the research was conducted in the absence of any commercial or financial relationships that could be construed as a potential conflict of interest. The reviewer GC declared a past co-authorship with two of the authors AG and JMD-G to the handling editor.
